# Neutrophil Progenitors Function as a Mechanistic Link Between Sleep Disruption and Heart Disease

**DOI:** 10.1097/HS9.0000000000000257

**Published:** 2019-05-22

**Authors:** Michael D. Milsom

**Affiliations:** 1 Division of Experimental Hematology, German Cancer Research Center (DKFZ), Heidelberg Germany; 2 Heidelberg Institute for Stem Cell Technology and Experimental Medicine (HI-STEM), Heidelberg, Germany.

Achieving sufficient good quality sleep is essential for maintaining physical and mental well-being, with sleep disruption being associated with a host of pathological disorders. Cardiovascular disease is one of the most prominent serious conditions whose incidence is linked to abnormal sleep patterns. While it is completely intuitive that lack of sleep can predispose one to illness, the molecular mechanisms that are responsible for such pathological consequences have not been well explored. In a recent manuscript published in Nature, McAlpine and colleagues describe the impact of sleep fragmentation on murine hematopoiesis and how the resulting impact on myeloid biology may comprise a causal molecular link with atherosclerosis.^1^

In order to interrogate potential mechanistic links between cardiovascular disease and sleep deprivation, this study first made use of an atherosclerosis-prone genetic mouse model that harbors a targeted deletion of the lipid transporter apolipoprotein E (*Apoe*^−/−^), resulting in: poor lipoprotein clearance; accumulation of cholesterol in the blood; and development of atherosclerotic plaques.^2^ When *Apoe*^−/−^ mice were subjected to sleep fragmentation, they developed larger plaques that contained a significantly higher proportion of myeloid cells compared to controls, suggesting that this would be a valid model to explore the etiology of this disease. Analysis of blood and bone marrow in both *Apoe*^−/−^ and wild-type mice demonstrated increased myeloid-biased hematopoiesis as a result of sleep deprivation, which correlated with the observation that leukocyte numbers are elevated in humans subject to sleep deficit. Since the hypothalamus is responsible for the production of several sleep-regulating proteins, its function was assessed in mice subject to sleep fragmentation, revealing decreased expression of the neuropeptide hypocretin. In humans, low serum levels of hypocretin are associated with increased risk of heart disease and obesity, suggesting that this could be a major mediator of the link between sleep interruption and heart disease. Importantly, analysis of hematopoiesis in hypocretin knock-out mice (*Hcrt*^−/−^) revealed a similar overproduction of monocytes and neutrophils and bone marrow myeloid progenitor expansion to that observed in sleep-fragmented mice, indicating a causal relationship. Transplantation of wild-type bone marrow into *Hcrt*^−/−^ mice also resulted in heightened hematopoiesis, reinforcing the hypothesis that compromised production of hypocretin in the hypothalamus can promote abnormal hematopoiesis in the bone marrow. In line with this data, combination of the *Hcrt*^−/−^ and the *Apoe*^−/−^ genotypes resulted in larger atherosclerotic lesions with greater levels of leukocyte recruitment than observed in control mice with the single *Apoe*^−/−^ mutation.

To delve into the molecular mechanism that links these processes, the authors next used a mouse GFP knock-in reporter line at the locus of the hypocretin receptor, *Hcrt1*, in order to establish that a subset of bone marrow pre-neutrophils expressed high levels of the receptor and were therefore likely to be responsive to hypocretin. Importantly, when such cells were isolated from *Hcrt*^−/−^ mice, they were found to express elevated levels of colony stimulating factor-1 (CSF1) compared to analogous cells from wild-type mice, implying a potential mediator of increased myelopoiesis in the setting of decreased hypothalamic hypocretin. In line with this observation, sleep fragmentation was also found to result in elevated production of CSF1 in the bone marrow and transplantation of *Hcrt1*^−/−^ bone marrow cells into wild-type recipients demonstrated increased myelopoiesis along with elevated levels of CSF1.

**Figure 1:**
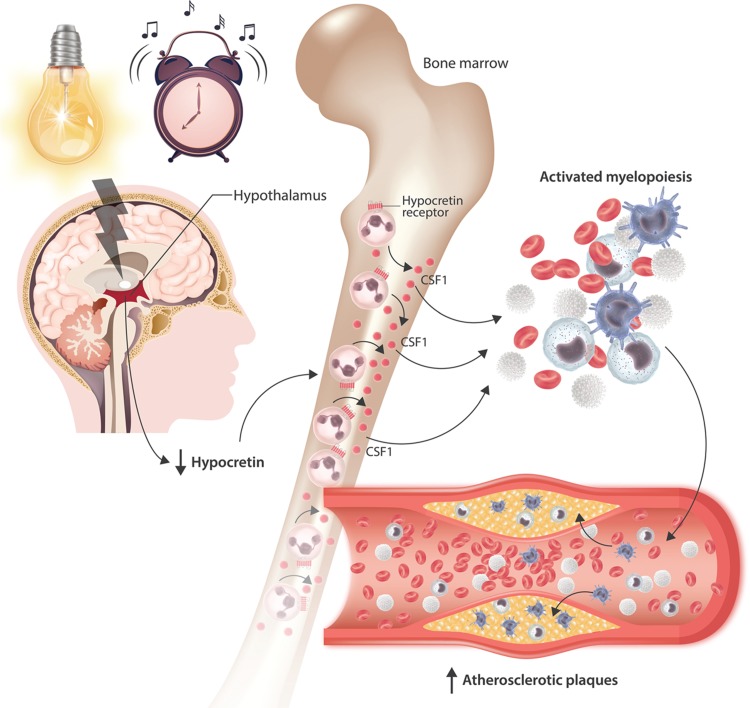
Schematic overview of the mechanism linking sleep disruption with atherosclerosis, as proposed by McAlpine et al. Sleep disruption leads to decreased production of hypocretin by the hypothalamus. In the bone marrow, a population of neutrophil progenitors that express the hypocretin receptor secrete increased levels of colony stimulating factor-1 (CSF1) as a result of decreased activity of this receptor, resulting in activated myelopoiesis. Recruitment of myeloid cells to atherosclerotic plaques is increased under these conditions.

Finally, the authors sought to complete the mechanistic link between sleep fragmentation, decreased hypothalamic hypocretin, increased production of CSF1 by bone marrow pre-neutrophils, and the generation of atherosclerotic plaques. Firstly, they used a different atherosclerosis-prone mouse model, which harbors a deletion of the low-density lipoprotein receptor (*Ldlr*^−/−^).^3^ These *Ldlr*^−/−^ mice were transplanted with either wild-type or *Csf1*^−/−^ bone marrow and were then subject to sleep fragmentation. Recipients of wild-type bone marrow demonstrated increased myelopoiesis and elevated bone marrow levels of CSF1 and developed large atherosclerotic plaques. In contrast, mice receiving the *Csf1*^−*/*−^ graft had a reduction in all of these properties, establishing that bone marrow production of CSF1 is a critical facilitator of both atherosclerosis and increased myelopoiesis as a result of sleep fragmentation. Secondly, the authors performed a second type of rescue experiment in order to firmly establish hypocretin as a mediator of this phenotype. Thus, *Apoe*^−/−^ mice were subject to sleep fragmentation as before, but were implanted with mini-osmotic pumps to deliver hypocretin into the periphery for a period of 8 weeks. Such mice had lower levels of circulating myeloid cells, decreased myeloproliferation and CSF1 in the bone marrow, and developed smaller atherosclerotic plaques (Fig. 1).

Taken together, this study provides novel molecular insight into the process via which sleep disruption can lead to cardiovascular disease as a result of dysregulation of hematopoiesis. Clearly, these findings are of potential translational relevance, both in terms of identifying biomarkers that might predict risk of cardiovascular disease in individuals with a history of chronic sleep disruption, and in terms of identifying potential novel targets that might facilitate therapeutic intervention in this process. One outstanding mechanistic link relates to whether the increased myelopoiesis observed in this model is, in itself, sufficient to facilitate the enhanced formation of atherosclerotic plaques. Alternatively, it remains to be seen whether the functional properties of CSF1-activated monocytes and neutrophils are altered in a way as to make them more prone to be recruited to such lesions and to more effectively contribute towards plaque formation. If so, it may be that this mechanism is more broadly applicable to the pathology of other diseases, such as those that are mediated by an excessive inflammatory innate immune response. It will also be interesting to determine whether chronic pharmacological induction of pseudo-normal sleep patterns by chronic ingestion of “sleeping tablets”, is also associated with a similar atheroscleric mechanism.
